# Sample Preparation and Extraction in Small Sample Volumes Suitable for Pediatric Clinical Studies: Challenges, Advances, and Experiences of a Bioanalytical HPLC-MS/MS Method Validation Using Enalapril and Enalaprilat

**DOI:** 10.1155/2015/796249

**Published:** 2015-03-19

**Authors:** Bjoern B. Burckhardt, Stephanie Laeer

**Affiliations:** Institute of Clinical Pharmacy and Pharmacotherapy, Heinrich-Heine-University, 40225 Düsseldorf, Germany

## Abstract

In USA and Europe, medicines agencies force the development of child-appropriate medications and intend to increase the availability of information on the pediatric use. This asks for bioanalytical methods which are able to deal with small sample volumes as the trial-related blood lost is very restricted in children. Broadly used HPLC-MS/MS, being able to cope with small volumes, is susceptible to matrix effects. The latter restrains the precise drug quantification through, for example, causing signal suppression. Sophisticated sample preparation and purification utilizing solid-phase extraction was applied to reduce and control matrix effects. A scale-up from vacuum manifold to positive pressure manifold was conducted to meet the demands of high-throughput within a clinical setting. Faced challenges, advances, and experiences in solid-phase extraction are exemplarily presented on the basis of the bioanalytical method development and validation of low-volume samples (50 *μ*L serum). Enalapril, enalaprilat, and benazepril served as sample drugs. The applied sample preparation and extraction successfully reduced the absolute and relative matrix effect to comply with international guidelines. Recoveries ranged from 77 to 104% for enalapril and from 93 to 118% for enalaprilat. The bioanalytical method comprising sample extraction by solid-phase extraction was fully validated according to FDA and EMA bioanalytical guidelines and was used in a Phase I study in 24 volunteers.

## 1. Introduction

For the last years both competent authorities, the US Food and Drug Administration (FDA) and the European Medicines Agency (EMA), force the development of high quality child-appropriate medications and intend to improve the availability of information on the pediatric use. Due to the current lack of sufficient evidence-based pharmacotherapy in children, sophisticated clinical investigations in all pediatric age groups (particularly in neonates and infants) are required to overcome this drawback.

Unfortunately, most bioanalytical assays are not yet tailored to meet current ethical and analytical burdens for research in children. Although the blood sample volume for determination of drug concentration is limited to microliters, it is essential for a valuable determination in pediatric patients to keep the calibration range as broad as or even broader than in assays applied in adult studies. HPLC-MS/MS is a predestinated analytical technique that appears to be the most suitable to deal with small sample volumes obtained from children and is linked with high selectivity for the quantification of the analytes of interest in diverse biological matrices. However, both the chromatographic equipment and the mass spectrometer (MS) encounter problems caused by the matrix. For example, the lifetime of the HPLC column is reduced if the sample purification is insufficient. Additionally, the detection by MS is susceptible to matrix effects leading to ionization suppression or enhancement [1, 2]. The matrix has a profound impact and restrains a precise quantification especially at the lower concentration levels. This ends up in nonrobust methods that do not encompass the broadest calibration range possible.

Therefore, the role played by proper sample preparation and extraction is important to overcome and control the interference caused through the biological matrices. A sophisticated sample clean-up removes material that chromatographically interferes with the analyte, enables appropriate recoveries, and erases matrix compounds that shorten column lifetime and affect the detection by MS. Attempts to simplify sample preparation and to reduce the preparation time ended in the awareness that this process accounts for less accuracy and precision in quantification [3]. Biological fluids like plasma, serum, urine, and saliva present a varying composition of, for example, lipids, proteins, electrolytes, cells, coeluting metabolites, impurities, and degradation products. All of these components might interfere with the analyte of interest. To reduce this interference several approaches had been developed in the past. Commonly used extraction techniques are protein precipitation (PPT), liquid/liquid extraction (LLE), and solid-phase extraction (SPE).

The purpose of the present work was to illustrate the importance of sample preparation exemplified by solid-phase extraction for the bioanalytical method development of low-volume assays for pediatric studies according to international agency guidelines. Using the method validation of enalapril, enalaprilat, and benazepril (internal standard), the encountered challenges and advances in sample preparation and solid-phase extraction as well as their effects on bioanalytical method validation of small volume samples were emphasized.

## 2. Methods

### 2.1. Material

The drug substances enalapril maleate CRS and enalaprilat dihydrate CRS (both European Pharmacopoeia Reference Standards) were purchased at the European Directorate for the Quality of Medicine & Healthcare (Strasbourg, France). Benazepril hydrochloride (≥ 98%, HPLC) and ethyl acetate (100% p.a.) were obtained from Sigma-Aldrich (Seelze, Germany). Methanol (HiPerSolv Chromanorm HPLC grade), water (super gradient grade), and acetone (AnalaR Normapur) were purchased from VWR (Germany). Alternative supplier of methanol (HPLC grade) was Fisher Scientific (Loughborough, United Kingdom). Formic acid (98–100% p.a.) was delivered by AppliChem (Gatersleben, Germany). Ammonium formate (99%, HPLC grade) was obtained from Fluka (Seelze, Germany). Blank human serum was provided by employees of the Institute of Clinical Pharmacy and Pharmacotherapy (Düsseldorf, Germany). Oasis 96-well plates (30 and 10 mg) and XBridge BEH C18 3.5 *μ*m columns (3.0 mm × 150 mm) were obtained from Waters (Eschborn, Germany).

### 2.2. Preparation of Standard and Quality Control

Stock solutions of enalapril, enalaprilat, and benazepril (internal standard) were prepared at 0.10 mg/mL in methanol. These stock solutions were diluted with water to obtain working solutions with 10 *μ*g/mL enalapril and enalaprilat as well as 166 ng/mL benazepril. For the calibration curve, blank serum was spiked with the analytes of interest and serially diluted. The final calibration range of the mass spectrometry was 0.2–200 ng/mL enalapril and 0.18–180 ng/mL enalaprilat. Quality control (QC) samples were independently prepared at four concentration levels over the whole calibration range (LLOQ, low, medium, and ULOQ).

### 2.3. Sample Preparation, Extraction, and Scale-Up

Based on preliminary investigations on the degree of sample dilution prior to solid-phase extraction a dilution ratio of 1 : 23 using water led to a robust method with high recovery rates for all analytes of interest.

Solid-phase extraction was chosen for the extraction process of the biological fluid owing to the superior purification performance if compared to protein precipitation or liquid-liquid extraction. Based on the compound properties of enalapril and enalaprilat, strong mixed-mode ion exchangers were chosen as sorbent material for the purification. Both a cation exchanger that interacts with the carboxylic acid groups and an anion exchanger that binds with the amino group of all above-mentioned substances were evaluated on their applicability (Oasis MCX and MAX material). The SPE protocol included a conditioning step utilizing methanol to enable optimal wetting of the cavernous sorbent material. For the subsequent equilibration step, different pH values and acids in aqueous solutions were tested to warrant for best interaction conditions prior the aqueous sample was loaded into the cavity. Namely, 2% formic acid (v/v), 4% phosphoric acid (v/v), 0.2 N hydrochloric acid, and pure water were evaluated. To purify the sample as much as possible, washing steps from hydrophilic to lipophilic properties were evaluated (water, 2% formic acid, hydrochloric acid, methanol, isopropanol, acetone, ethyl acetate, and mixtures of the aforementioned). Elution was assessed by acidified methanol for MAX sorbent material and ammonium in methanol for MCX sorbent material. The kind of acid (or base), its concentration, and the corresponding elution volumes were investigated to obtain all analytes within one fraction by reducing the coeluted residual matrix to a minimum.

**Figure 1:**
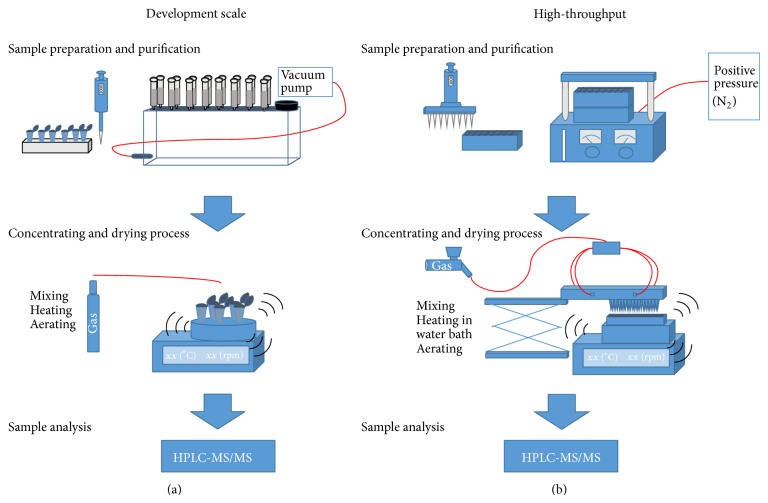
Scale-up process and optimization steps to establish a high-throughput approach of all bioanalytical assays utilizing solid-phase extraction. On the left side the development approach of sample preparation and purification is illustrated (a). The solid-phase extraction is performed by cartridges using the vacuum manifold. By contrast the scale-up is shown on the right side (b). Samples were prepared in 96-well approaches utilizing multichannel pipettes. The purification is conducted on a positive pressure manifold with 96-well plates. For the drying process the applied thermomixer was modified by a heatable water bath and a special drying top frame to deal with the deep-well collection plates (in-house development).

To meet current demands in sample throughput within a clinical study, the scale-up to offline positive pressure extraction was conducted. The switch to 96-well format came along with a changeover from vacuum extraction to positive pressure extraction. SPE-formats with higher amounts of SPE cavities per plate were commercially not available. For the critical steps of sample load, washing, and sample elution, the positive pressure was kept between 1 and 2 psi to warrant an intensive interaction between analyte and sorbent material as well as reproducible flow rate.  Figure 1 illustrates the corresponding scale-up.

### 2.4. Chromatographic and Mass Spectrometric Conditions

The utilized modular HPLC system (Shimadzu Deutschland GmbH, Duisburg, Germany) consisted of a controller SCL-10Avp, two separate pumps LC-10ADvp, three-channel online degasser DGU-20A^3^ prominence, autosampler SIL-10ADvp, and a column oven (L-2300, VWR/Hitachi). For the separation of enalapril and enalaprilat an XBridge BEH C18 3.5 *μ*m column (3.0 mm × 150 mm) was used. After injection of 10 *μ*L sample solution (methanol/water 40 : 60, v/v) the samples were separated under gradient conditions within 6-minute run time utilizing a methanol/water mixture (40 : 60, v/v) buffered with formic acid (1%, v/v) and ammonium formate (2 mM). The applied gradient started with 40% of methanol and increased stepwise after 0.5 minute to 60% and after 1 minute to 80%. Between 1 and 4 minutes the amount of methanol was continuously increased to 95% and reduced at 4.5 minutes to 40% of methanol again. It stayed at this level till the end of the run time. The flow rate was 0.4 mL/min and the column temperature was maintained at 50°C which resulted in a moderate back pressure of 125 bar. Triple-quadrupole tandem mass spectrometric detection was performed on an Applied Biosystems SCIEX API 2000 (Applied Biosystems/MDS SCIEX, Concord, Canada) with an electrospray ionization (ESI) interface running in positive ionization mode. The device screened the transitions channels 377.2 to 234.2* m/z* (enalapril), 349.1 to 206.1* m/z* (enalaprilat), and 425.3 to 351.2 m/z (benazepril) in multiple reaction monitoring (MRM) mode. All dwell times were set to 250 ms.

### 2.5. Validation

The bioanalytical method was fully validated according to current FDA and EMA bioanalytical guidelines as a quantitative confirmatory method in terms of linearity, specificity, accuracy, precision, recovery, matrix effect, and stability [4, 5]. A main focus during validation was on the extraction process. The latter was in particular validated on recovery of the extraction process and absolute plus relative matrix effect. Additionally, extraction process efficiency and interference caused by hyperlipidemic and hemolyzed samples were evaluated.

The ratio of peak area of serum spiked with analyte prior to solid-phase extraction (Area_*A*_) with the peak area of blank serum spiked with analyte after the extraction (Area_*B*_) yielded the recovery of the assay. Recovery was determined at four concentration levels with five replicates per level. Calculation was performed as follows:

(1) $$\begin{array}{c} \text{RE}\left[\%\right]=\left(\left.\frac{{\text{Area}}_{A}}{{\text{Area}}_{B}}\right)\right.*100, \end{array}$$

*where *RE* = recovery; *Area_*A*_ *= peak area of serum spiked with analyte prior to extraction; *Area_*B*_ *= peak area of blank serum spiked with analyte after the extraction.*

Although the blood composition and pH value are strongly controlled and vary only slightly within a healthy subject, the overall consequence of all compounds in a biological sample matrix contributes to the matrix effect that alters the accurate and precise determination of the analyte of interest. Therefore, a suitable sample preparation and purification by SPE contributes extremely to a robust method being less-sensitive to the effect. Apart from broadly investigated absolute matrix effect, the investigation of the relative matrix effect appears in this contect more relevant for precise and robust methods for the analysis of biological samples [2].

Calculation of the absolute matrix effect was conducted by calculating the ratio of the peak area of extracted human serum postspiked with analyte (Area_*x*_) to the peak area of the analyte in the same concentration dissolved in mobile phase (Area_*y*_). The absolute matrix effect was determined at four concentration levels with five replicates per level. The absolute matrix effect was calculated according to the equation by Matuszewski et al. [2]:

(2) $$\begin{array}{c} \text{ME}\left[\%\right]=\left(\left.\frac{{\text{Area}}_{x}\;}{{\text{Area}}_{y}}\right)\right.*100, \end{array}$$

*where *ME* = matrix effect; *Area_*x*_ *= detected peak area of extracted human serum postspiked with analyte; *Area_*y*_ *= detected peak area of dissolved analyte in mobile phase.*

To distinguish between ion suppression and ion enhancement caused by the matrix, the calculated matrix effect was subtracted by 100. A value < 0 indicated ion suppression caused by the matrix while ion enhancement was present if the calculated value was > 0.

According to international guidelines, the absolute matrix effect should be evaluated but is not limited to a certain range. However, the applicant needs to be able to estimate the effect of the matrix on the assay's performance.

By contrast the European Medicines Agency provides a guidance mentioning fixed acceptance criteria for the relative matrix effect. The intersubject variability of the internal standard normalized relative matrix effect (IS-ME) of processed samples should be maximum 15% and is expressed as coefficient of variation (CV) [4]. The calculation of the latter was done by evaluation of the individual IS-normalized matrix effect being defined as the matrix factor of the analyte divided by the matrix factor of the IS. The matrix factor represents the ratio of the peak area in the presence and the absence of the matrix of the corresponding substance. The CV of the IS-normalized matrix effects of seven subjects was used to assess the relative ME. The relative matrix effect was evaluated at 0.39 ng/mL enalapril and 0.35 ng/mL enalaprilat (low concentration level) as well as at 200 ng/mL enalapril and 180 ng/mL enalaprilat (ULOQ). Per concentration level three replicates per subject were analysed:

(3) IS-ME%  =Peak area of analytePresence of matrixPeak area of analyteAbsence of matrix    ∗Peak area of ISPresence of matrixPeak area of ISAbsence of matrix−1∗100,

*where *IS-ME* = internal standard normalized matrix effect.*

To calculate the process efficiency of the solid-phase extraction the following equation by Taylor [1] was used:

(4) $$\begin{array}{c} \text{PE}\left[\%\right]=\text{RE}\left[\%\right]*\frac{\left(100-\text{ME}\left[\%\right]\right)}{100}, \end{array}$$

*where *PE* = process efficiency; *RE* = recovery; *ME* = absolute matrix effect.*

Further validation parameters, as listed in the international bioanalytical guidelines, were investigated as follows: linearity was evaluated by measuring freshly spiked human serum with enalapril and enalaprilat at 11 concentration levels. The bioanalytical method was evaluated on four different days by four different runs on accuracy and precision. Therefore, five independently prepared quality control samples were assessed on four concentration levels (enalapril: 0.2, 3.13, 25, and 200 ng/mL; enalaprilat: 0.18, 2.81, 22.5, and 180 ng/mL) per run. The precision was determined by ANOVA while the accuracy was described by percentage deviation of the mean value to the nominal values of each concentration level. A maximum deviation of ±15% (±20% at the LLOQ) was regarded as acceptable [4]. The selectivity was assessed by check for interaction caused by 7 human serum samples spiked with 11 common comedications. The long-term stability of the drugs was evaluated at −80°C for at least 60 days, short-term stability conducted at 20°C for 24 h, and autosampler stability for 24 h also at 20°C. Additionally, the stability of the dried eluate after sample extraction was investigated at −20°C for 24 h.

### 2.6. Application

The high-throughput approach was applied to a Phase I study in 24 healthy volunteers. Both urine and serum samples were withdrawn and analyzed after administration of 10 mg enalapril maleate. With focus on sample extraction, the applicability was evaluated on the time required to run the extraction, the occurrence of any clotting of the cavity during extraction, and the goodness of extraction by checking for any shift in retention time in samples of different volunteers and different sample conditions (e.g., hemolyzed samples).

## 3. Results and Discussion

### 3.1. Sample Preparation

**Figure 2:**
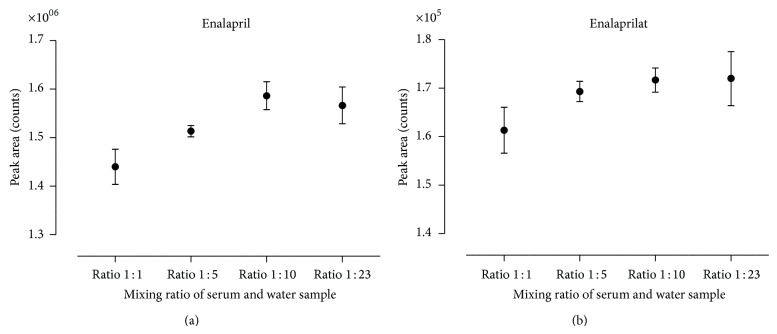
Comparison of mixing ratios of serum and water on resulting peak areas. The detected peak areas of enalapril (a) and enalaprilat (b) of purified serum samples are presented. The mixing ratio was varied between 1 : 1 and 1 : 23. Each determination was conducted by three independently prepared quality control samples. The mean and corresponding standard deviations are shown.

Preliminary investigations on the degree of sample dilution had been shown to influence the extraction performance and accounted highly for a robust method with high recovery. Investigations on suitable sample dilution solvents (formic acid, phosphoric acid, hydrochloric acid, and water) and their mixing ratio with the sample itself were conducted. To determine the best suitable mixing ratio of acids or pure water, the ratio was varied between 1 : 1 and 1 : 23. The conducted investigations on the most appropriate dilution solvent showed that water is sufficient if high dilution factors were applied. By increasing the mixing ratio, the detected peak areas of enalapril and enalaprilat increased in parallel (Figure 2). A mixing ratio of 1 : 10 and 1 : 23 worked best with regard to sample recovery. The highest dilution ratio resulted in a total sample volume of about 1.2 mL. Owing to the maximum capacity of a cavity (~1.4 mL), a higher degree of dilution is not recommended for routine. It increases the risk of carryover and rises the likelihood of sample mix-up as the sample solution needs to be pipetted at least in two parts into the cavity. The final composition of the diluted sample solution consisted of 50 *μ*L serum being mixed with 5 *μ*L benazepril working solution (IS) and 1100 *μ*L water.

### 3.2. Sample Extraction

Specifically, if small biological sample volumes like in pediatric research require purification, the sample extraction plays a particular role. Commonly, protein precipitation (PPT), liquid/liquid extraction (LLE), and solid-phase extraction (SPE) are applied as extraction techniques. PPT is a fast and simple approach but works best only in protein-rich matrices such as whole blood, plasma, or serum. Nevertheless, the PPT is nonselective and does not remove matrix interferences other than proteins. In the investigations by Dams et al., PPT in combination with LC-MS/MS had the greatest matrix effect amongst the investigated purification approaches [3]. It is a useful and fast technique to optimize lifetime of the equipment but does not increase the analytical sensitivity which is important for low-volume LC-MS/MS assays. LLE allows separation of analytes of interest from proteins and other hydrophilic components, but if emulsions are formed, the separation of the organic solvent becomes difficult and might result in incomplete and various-analyte diffusion. Jessome and Volmer emphasized the cumbersome sample preparation by LLE and LC-MS/MS [6]. Especially the complex adjustment of the pH value for the transfer in the organic phase, extraction of highly polar substances and necessary multiple extraction steps are some challenges faced. In particular for high-throughput analytics—as it is useful in clinical study approaches—the LLE is not the first choice. Consequently, SPE was selected owing to its superior purification properties and flexibilities in extraction protocols to cope with diversity of analytes, purifications solvents, and biological fluids.

The selected drug combination (calculated log⁡*P* values of enalapril: 2.5; enalaprilat: −0.9; benazepril: 3.5) carried the risk of improper binding to the sorbent due to Coulomb repulsion, losing hydrophilic analytes during too extensive and not well-balanced washing steps or by incomplete recovery of the more lipophilic compounds from the sorbent material during elution. All this may be attributed to low recovery or bad reproducibility which in turn narrows the calibration range, because especially lower concentrations might not conform to international guideline requirements. Available sorbent phases characterized by different interaction possibilities (van der Waals forces, ionic interaction, etc.) and different amounts of sorbent per cavity and the high flexibility in the SPE protocol on how intensive the purification of sample needs to be conducted represent some of many useful tools to overcome those drawbacks.

**Figure 3:**
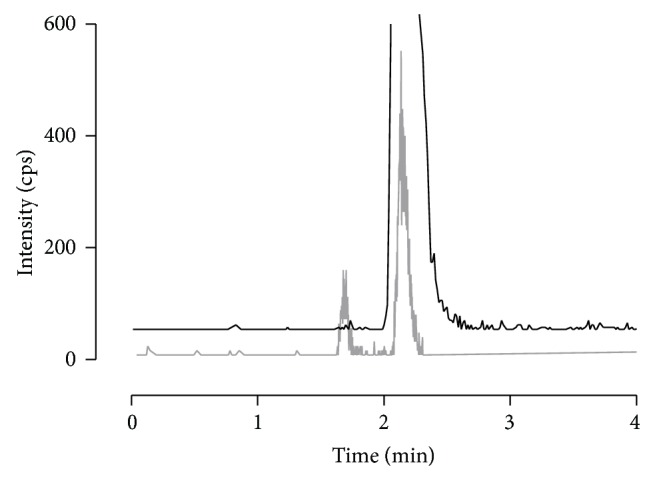
Determined split peak in serum with the transition 349.1 → 206.1* m/z* during method development. The split peak was measured on several HPLC columns after SPE purification by Oasis MCX. In grey, the ion count of a low enalaprilat concentration in serum is shown that clearly identifies the split peak. As reference, the enalaprilat standard solved in mobile phase is presented by the black line without any split peak (base line is nudged to prevent overlap).

First extraction attempts were undertaken by utilizing Oasis MCX. This polymeric material is characterized by a strong cation exchanger (on sulfonic acid base) binding the carboxylic acids groups of the analytes of interest. However, purified samples showed a split peak for the selected transition of enalaprilat if determined by HPLC-MS/MS. The corresponding chromatograms revealed a peak occurring at an earlier retention time plus a second peak at the expected retention time of the compound (Figure 3).

The peak area and intensity of the first peak did not alter with different enalaprilat concentrations per sample and accounted therefore most likely for a residual serum matrix component. It was not possible to erase the peak neither by thought-out SPE nor by any number of different LC gradients or by different HPLC columns featured with opposed chromatographic properties (Atlantis T3, XBridge, and XSelect). The checks for contamination in mobile phase, autosampler, or solutions for SPE were additionally negative. At the lowest concentration level of the calibration curve, the first peak and the one of enalaprilat were comparable in shape, intensity, and peak area. This phenomenon carried the risk of less robustness if automated integration of the chromatogram is preferred. After also scanning for the second most intense transition of enalaprilat (349.1 → 303.1* m/z*), clarity was brought to question as the first peak did not belong to enalaprilat. However, quantification with two transitions would have resulted in a higher LLOQ which was undesired as very low concentration levels are expected in the scheduled pediatric studies. Therefore, the sorbent material of the SPE was changed from cation exchanger to strong anion exchanger (MAX) and a new extraction protocol was developed. This brought success to the method, as the compound with the same transition as enalaprilat (349.1 → 206.1* m/z*) could be detached by SPE and consequently the split peak was removed.

**Figure 4:**
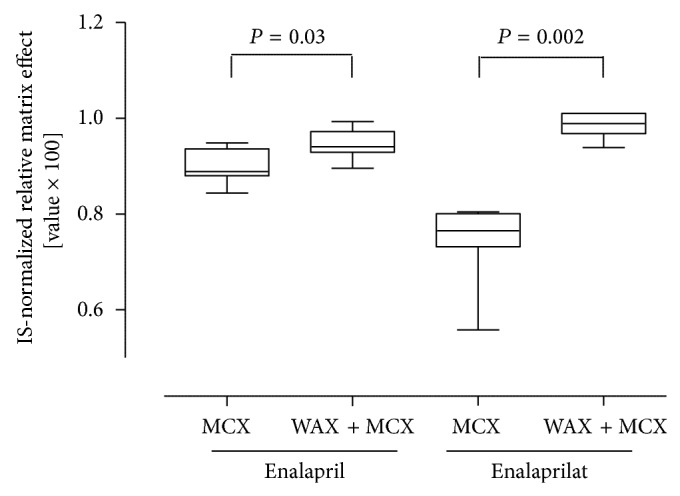
Comparison of different extraction methods on the internal standard normalized matrix effect. The SPE extraction by Oasis MCX is compared to the two-step extraction by Oasis WAX + MCX. Each boxplot describes median, 25th, 75th percentile + 10th, and 90th percentile as whisker. *N* = 9 measurements per boxplot. Statistical analysis was performed by a Mann-Whitney-*U* test (two-tailed *P* value).

At this stage an excuse to an already established and fully validated extraction method for the same drug entities in urine (amongst others) is made to introduce another useful approach to purify the sample matrix, to reduce the relative matrix effects, and to meet the current EMA bioanalytical guideline [4]. A two-step solid-phase extraction by a weak anion exchanger followed by a strong cation exchanger significantly reduced the internal standard normalized matrix effect compared to the purification by MCX only (Figure 4). The more hydrophilic the compound was the merrier the improvement of the matrix effect was pronounced. However, it expounds how diverse the several biological fluids are and emphasizes the high required effort in method development to reduce the relative matrix effect. By applying the final two-step extraction the following results for the relative matrix effect were obtained: at the LLOQ the CV was 4.04% and 6.62% for enalapril and enalaprilat, respectively. A CV of 1.26% for enalapril and 1.25% for enalaprilat was calculated at the ULOQ and was therefore well within the EMA requirements of 15% [7]. This bioanalytical urinary method was fully validated according to the strictest validation parameters of EMA and FDA bioanalytical guidance [7].

**Figure 5:**
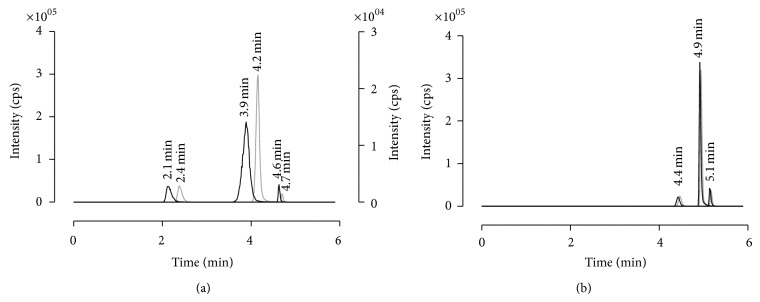
Multiple reaction monitoring scan chromatograms of enalapril, enalaprilat, and benazepril. The left graph illustrates the shift in retention time detected by comparing the analytes solved in mobile phase versus the analytes in purified matrix during first extraction attempts. Right graph shows the final chromatogram by comparing also the analytes in mobile phase versus analytes in purified serum. A timely shift in retention time was not detectable anymore. MRMs: 377.2 → 234.2* m/z* (enalapril), 349.1 → 206.1* m/z* (enalaprilat), and 425.3 → 351.2* m/z* (benazepril). The black line identifies the extracted serum samples and the grey line illustrates the drug substances dissolved in mobile phase. Cps: counts.

At an early development stage of the extraction protocol in serum at which the extraction was not methodologically sound, we encountered some deviation in retention time when comparing the chromatograms of analyte solved in mobile phase to extracted analyte in serum (Figure 5). By increasing the amount of organic washing steps as well as the elution force of the used organic solvents (methanol, acetone, and ethyl acetate), this timely shift could be eliminated (Figure 5). Even the intensities and peak areas of both, the analyte in mobile phase and the analyte in purified serum, were finally comparable. This pointed out the gained high degree of sample purification and accounted for a very limited signal suppression by the residual matrix compound in the final extract. However, it illustrates that matrix effects not only affect the signal intensities of the mass spectrometer but can also alter the chromatographic performance. A comparable effect was recently published by Fang et al. [8].

**Figure 6:**
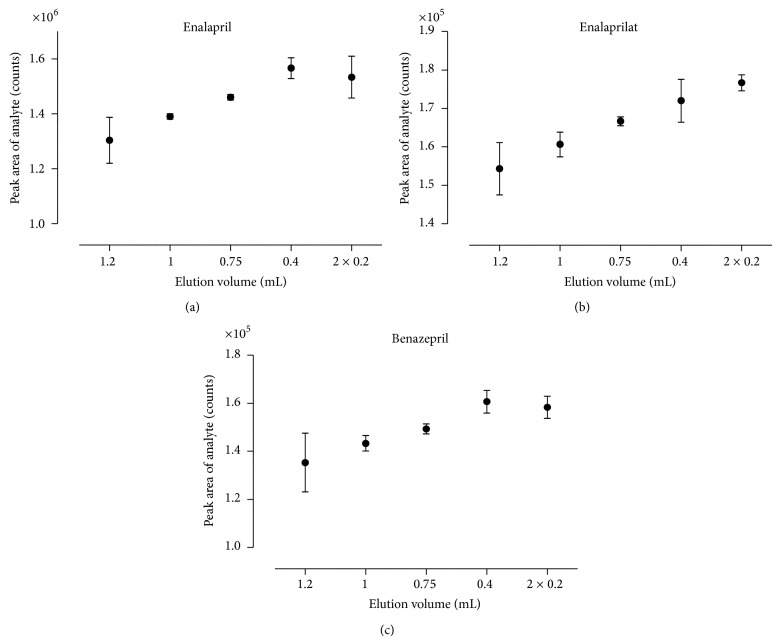
Effect of elution volume on peak area of the analytes of interest. The peak areas of enalapril (a), enalaprilat (b), and benazepril (c) after elution are presented with different volumes of 2% formic acid in methanol. Each determination was conducted by three independently prepared quality control samples. The mean and corresponding standard deviations are shown.

Finally, the conducted investigations on the most appropriate elution solvent and its volume yielded 0.4 mL acidified methanol (2% formic acid, v/v). This elution step did not only served to elute the analytes of interest but also acted as a final step to fraction the analytes of interest and other interfering residual compounds. Hereby the choice and elution force of the solvent as well as the amount of solvent were investigated on their effect to attribute to a rugged, reliable, and selective protocol. As illustrated in Figure 6, there was a reciprocal relationship between the elution volume of acidified methanol and the detected peak areas of the analytes of interest. The obtained smaller peak areas—after the sorbent material was eluted with higher volumes of acidified methanol—might be explained by the fact that more interfering matrix was coeluted. The interference induced by the matrix led to ion suppression and smaller peak areas.

The final protocol for the serum method was destined to the following approach: the samples were extracted with Oasis MAX solid-phase extraction cartridges (10 mg, 1 mL)—a mixed mode, reverse-phase, and strong anion exchanger (on quarternary amine base). The MAX 96-well plates chosen allowed for high sample throughput and were primed with 1 mL of formic acid in methanol (2%, v/v) followed by an equilibration step with 1 mL water. On the one hand, this aqueous step prior to sample load ensured that, for example, no denaturation and therefore clotting of sample matrix on the sorbent material happened. On the other hand, it offered the best interaction conditions with the sorbents. After the sample mixture was loaded into the cartridges and passed, the sorbent of the cartridges was washed by 1.0 mL of water, 1.0 mL of methanol-acetone mixture (60 : 40, v/v), 1.0 mL of ethyl acetate, and 500 *μ*L of methanol. The increasing elution force of the investigated organic solvents was used mainly to wash out phospholipids that are known to be responsible for a large part of matrix effect in blood, serum, and plasma [9]. Finally the analytes were eluted from the cartridges once with 0.4 mL of formic acid in methanol (2%, v/v). The eluate was evaporated to dryness under a gentle stream of compressed air while shaking at 550 rpm at 40°C. The residue was reconstituted with 100 *μ*L of methanol and water (40 : 60, v/v).

### 3.3. Scale-Up of Sample Preparation and Extraction to 96-Well Setting

For reproducible and high-quality solid-phase extraction (SPE) with a high run-to-run consistency as desired for clinical studies, the switch from single cartridges by vacuum extraction to 96-well positive pressure extraction was conducted. This scaling presented the highest possible offline scaling for the used SPE material. SPE-formats with higher amounts of SPE cavities per plate were commercially not available. The conventional vacuum manifold had the disadvantage of irreproducible analyte recoveries due to variable processing times in the columns. For the highest possible reproducibility during extraction, the controlled and appropriate flow rate is much more essential than applying either vacuum or positive pressure. However, the positive pressure manifold had the advantage of being equipped with a monitor to check for the flow rate of the liquid. Specifically the sample load, washing, and elution step are known to be the most sensitive and critical steps regarding the flow rate. The exact adjustment of the flow rate of the liquids was important to generate a well-balanced setting of high reproducibility, best extraction speed and duration of sample extraction.

**Figure 7:**
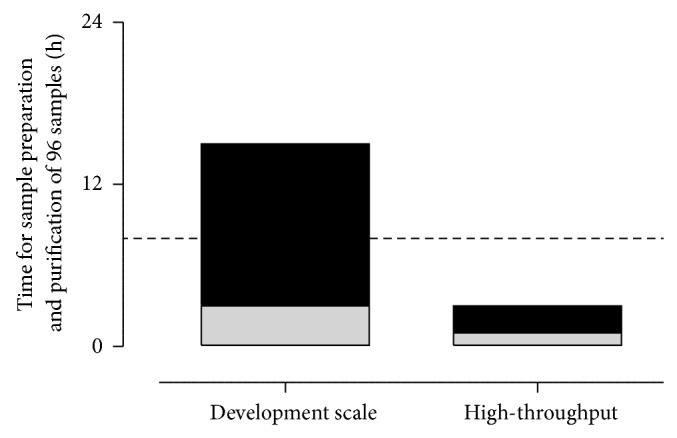
Comparison of required time for bioanalysis between development scale by applying single cartridges and the high-throughput approach with 96-well plate. The calculation bases on a sample amount of 96 samples. The black areas mark the required time for sample purification by solid-phase extraction and grey areas identify the time frame required for sample preparation. The dashed line represents one full working day (8 hours). By applying the high-throughput approach the sample preparation and purification is finalized within 2 hours while the same amount of samples is impossible to purify within one working day by one lab technician using the previous development scale.

The transfer from vacuum extraction to positive pressure not only enabled a semiautomated extraction but also highly increased the sample amount up to about thousand samples which can be purified per week by one laboratory technician. Since appropriate equipment for rapid and continuous drying was not commercially available, required equipment for the drying process was self-developed to suit best the laboratory preconditions and needs.  Figure 1 is enclosed illustrating the scale-up from single cartridges using vacuum technique to the positive pressure extraction in 96-well formate. As indicated in Figure 7, the scale-up is a mandatory step in method development if the assay will be applied to analyze hundreds or thousands of samples. In our positive pressure approach, it took about 2 hours from raw sample to sample preparation and extraction to the final sample solution ready to be determined by HPLC-MS/MS. In such a run, 96 samples could be prepared which required much more than one full working day (8 h) of one laboratory technician to prepare the same amount by the previously used vacuum manifold approach. The applied pressures of 1–3 psi were fully sufficient to ensure a continuous and appropriate flow rate of solvent/sample solution through the sorbent material. The scale-up enabled a nearly sixfold higher sample throughput.

### 3.4. Validation

The chosen calibration range of 0.2–200 ng/mL enalapril and 0.18–180 ng/mL enalaprilat, respectively, showed guideline-conforming linearity over all eleven concentration levels. Best fit of the linear regression was gained by 1/*x* ^2^ weighting for both analytes. No additional peaks above the guideline limits within ±0.3 minutes of the retention time of enalapril, enalaprilat, and benazepril were observed in the spiked serum samples with coadministered drugs (acetylsalicylic acid, aliskiren, ramipril, ramiprilat, candesartan, atenolol, bisoprolol, metoprolol, pantoprazole, and pravastatin). No interferences in blank samples of the different sources were detected and the signal-to-noise ratios of spiked to blank samples of all sources were above 5 : 1. Obtained results by one-way ANOVA for the intrarun precision ranged from 2.2 to 5.0% for enalapril and from 4.9 to 18.0% for enalapril. These precision results conformed all to the current bioanalytical guidelines. Mean accuracy results of the quality control samples were likewise within the guideline requirements of ±15% at all concentration levels (at the LLOQ ±20%). Hemolyzed blood as well as hyperlipidemic blood samples had no detectable influence on the specific MS-channels of enalapril, enalaprilat, and IS, respectively. Obtained stability results of enalapril and enalaprilat for long-term storage (−80°C, 60 days), for short-term storage (20°C, 24 h), and in the autosampler (20°C, 24 h) proved the drug stability. Additionally, both drug substances showed no significant degradation if they were stored as dried elution extract at −20°C for 24 h.

The effect of the matrix on the determination of enalapril and enalaprilat was evaluated at the LLOQ (0.2 ng/mL; 0.18 ng/mL), one low concentration (3.13 ng/mL; 2.81 ng/mL), one middle concentration (25 ng/mL; 22.5 ng/mL), and at the ULOQ (200 ng/mL; 180 ng/mL). By combining SPE and chromatographic separation the matrix effect was observably reduced in this setting, leading to an ion suppression of −8.9 to −19.8% for enalapril and of −7.2 ± 2.8% for the internal standard benazepril. The sample matrix had no or a slight ion enhancing effect on detection of enalaprilat. It ranged between −1.5 and 10.5%. All analytes were almost fully recovered from the sorbent of the mixed-mode anion exchanger, resulting in a process efficiency of 67 to 94% for enalapril, 95–119% for enalaprilat, and 71% for benazepril. Details are arranged in Table 1.

The relative matrix effects of the extracted serum samples at a low concentration level (0.39 ng/mL enalapril and 0.35 ng/mL enalaprilat, resp.) were 5.49% (CV) for enalapril and 12.56% for enalaprilat. At the ULOQ, coefficients of variation of 1.87% for enalapril and 8.96% for enalaprilat were evaluated for all seven different human sources. All findings complied with EMA bioanalytical guideline. Details are arranged in Table 2.

### 3.5. Application

**Figure 8:**
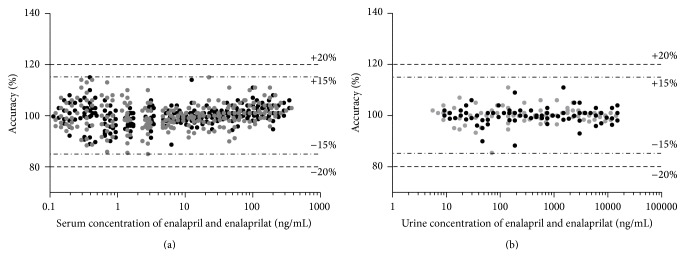
The plots show the accuracy results of 22 serum calibration curves and the accuracy results of 7 urinary calibration curves (each covering 11 concentration levels per drug substance) of enalapril (black) and enalaprilat (grey) used for the evaluation of the obtained results of the conducted Phase I study. Additionally the accuracy thresholds (dashed lines) according to FDA and EMA bioanalytical guidelines for all concentrations levels (±15%) and the LLOQ (±20%) are indicated.

For the Phase I study, in total approximately 1600 serum and 600 urinary samples were analyzed. No clotting of serum sample solution in any SPE cavity was noticed. A shift in the retention times of enalapril, enalaprilat, and benazepril in purified serum samples of 24 different sources was not detected during the analysis by HPLC-MS/MS and allowed for an automated intergation. Furthermore, hemolyzed samples did not affect the extraction run negatively and showed no significant interference during analysis. The determined pharmacokinetic results of the Phase I study will be used to apply a new marketing authorization and remain therefore confidential. However, during sample determination 22 calibration curves in serum and 7 in urine were required to quantify the drug concentration in the corresponding samples. Obtained results of the calibration curves on intra- and interrun accuracy proved the applicability of the established bioanalytical method comprising the good sample extraction and their suitable preparation.  Figure 8 shows the accuracy results for the serum and urine calibration curves.

Furthermore, these tailored low-volume assays will be applied to pediatric Phase II and III studies. The available pediatric study investigating the pharmacokinetics of enalapril and its active metabolite enalaprilat in hypertensive children was published by Wells et al. using a radioimmunoassay [10]. They found mean concentrations between 2 and 25 ng/mL enalaprilat. Lloyd et al. reported enalaprilat concentration values between 0.9 and 12.7 ng/mL in children with heart failure [11]. Both reported ranges are covered by the linear range of the assay presented and confirm its applicability for pediatric research. The required sample volume of 50 *μ*L serum for reliable determination appears additionally well suitable for clinical trials in all age groups, especially for neonates and newborns. The required sample volumes of published LC-tandem mass spectrometry assays on enalapril and enalaprilat range between 200 and 1000 *μ*L blood [12–17].

## 4. Conclusion

Using the example validation of the low-volume bioanalytical method of enalapril and enalaprilat, pitfalls as well as improvements of the extraction protocol were shown. The aim of an accurate and precise low-volume method encompassing a broad calibration range with very low limits of quantification was only gained by complex extraction protocols. The calibration range of the established assay covers reported enalapril and enalaprilat concentrations in pediatric patients and proves its applicability for pediatric research. It was shown that the undertaken efforts for a sophisticated extraction protocol utilizing solid-phase extraction resulted in a high recovery and high reduction of matrix effect. Controlling the latter—amongst others—warranted for a valuable and reliable bioanalytical method in serum. It allowed successful validation of a low-volume bioanalytical HPLC-MS/MS method according the FDA and EMA bioanalytical guidelines. The applicability of the high-throughput approach was proven by a clinical study in 24 volunteers.

## Figures and Tables

**Table 1:** Results for recovery, absolute matrix effect, and process efficiency.

	Concentration [ng/mL]	Absolute matrix effect ± S.D. [%]	Mean recovery ± S.D. [%]	Mean process efficiency [%]
Enalapril	0.195	−12.6 ± 11.9	77.1 ± 0.7	67.4
	3.13	−8.9 ± 2.2	103.5 ± 3.7	94.3
	25	−19.8 ± 4.4	102.9 ± 6.3	77.6
	200	−17.5 ± 3.5	92.0 ± 6.9	72.5

Enalaprilat	0.175	−1.5 ± 10.8	99.9 ± 7.8	98.4
	2.81	0.3 ± 3.7	118.3 ± 4.6	118.7
	22.5	10.5 ± 4.9	102.4 ± 4.7	113.2
	180	2.2 ± 3.5	92.9 ± 9.2	94.9

Benazepril	25	−7.2 ± 2.8	76.9 ± 2.3	71.3

Data compiled as mean or mean ± standard deviation (S.D.).

**Table 2:** Relative matrix effect obtained in seven different human sources.

Donor	Enalapril		Enalaprilat	
	0.39 ng/mL	200 ng/mL	0.35 ng/mL	180 ng/mL
Healthy adults				
29–86 years old				
♂ Donor 1	106.7	96.8	162.5	135.9
♂ Donor 2	99.1	95.7	122.1	108.2
♂ Donor 3	96.6	96.6	120.9	119.4
♀ Donor 4	111.1	97.6	132.1	126.6
♀ Donor 5	107.2	97.8	121.0	114.5
♀ Donor 6	98.8	94.5	123.1	112.1
♂ Donor 7	98.4	92.8	114.4	106.7

Mean value of normalized relative ME ± S.D. [%]	102.6 ± 5.6	96.0 ± 1.8	128.0 ± 16.1	117.6 ± 10.5
CV [%] within donors	5.49	1.87	12.56	8.96

ME: matrix effect; S.D.: standard deviation; CV: coefficient of variation.
